# Assessing effects of germline exposure to environmental toxicants by high-throughput screening in *C*. *elegans*

**DOI:** 10.1371/journal.pgen.1007975

**Published:** 2019-02-14

**Authors:** Nara Shin, Luciann Cuenca, Rajendiran Karthikraj, Kurunthachalam Kannan, Monica P. Colaiácovo

**Affiliations:** 1 Department of Genetics, Harvard Medical School, Boston, MA, United States of America; 2 Wadsworth Center, New York State Department of Health, Empire State Plaza, Albany, New York, United States of America; 3 Department of Environmental Health Sciences, School of Public Health, University at Albany, State University of New York, Albany, New York, United States of America; The University of North Carolina at Chapel Hill, UNITED STATES

## Abstract

Chemicals that are highly prevalent in our environment, such as phthalates and pesticides, have been linked to problems associated with reproductive health. However, rapid assessment of their impact on reproductive health and understanding how they cause such deleterious effects, remain challenging due to their fast-growing numbers and the limitations of various current toxicity assessment model systems. Here, we performed a high-throughput screen in *C*. *elegans* to identify chemicals inducing aneuploidy as a result of impaired germline function. We screened 46 chemicals that are widely present in our environment, but for which effects in the germline remain poorly understood. These included pesticides, phthalates, and chemicals used in hydraulic fracturing and crude oil processing. Of the 46 chemicals tested, 41% exhibited levels of aneuploidy higher than those detected for bisphenol A (BPA), an endocrine disruptor shown to affect meiosis, at concentrations correlating well with mammalian reproductive endpoints. We further examined three candidates eliciting aneuploidy: dibutyl phthalate (DBP), a likely endocrine disruptor and frequently used plasticizer, and the pesticides 2-(thiocyanomethylthio) benzothiazole (TCMTB) and permethrin. Exposure to these chemicals resulted in increased embryonic lethality, elevated DNA double-strand break (DSB) formation, activation of p53/CEP-1-dependent germ cell apoptosis, chromosomal abnormalities in oocytes at diakinesis, impaired chromosome segregation during early embryogenesis, and germline-specific alterations in gene expression. This study indicates that this high-throughput screening system is highly reliable for the identification of environmental chemicals inducing aneuploidy, and provides new insights into the impact of exposure to three widely used chemicals on meiosis and germline function.

## Author summary

The ever-increasing number of new chemicals introduced into our environment poses a significant problem for risk assessment. In addition, assessing the direct impact of toxicants on human meiosis remains challenging. We successfully utilized a high-throughput platform in the nematode *C*. *elegans*, a genetically tractable model organism which shares a high degree of gene conservation with humans, to identify chemicals that affect the germline leading to aneuploidy. We assessed chemicals that are highly prevalent in the environment in worms carrying a fluorescent reporter construct allowing for the identification of X chromosome nondisjunction combined with a mutation increasing cuticle permeability for analysis of low doses of exposure. Follow up analysis of three chemicals: DBP, permethrin and TCMTB, further validated the use of this strategy. Exposure to these chemicals resulted in elevated levels of DNA double-strand breaks, activation of a DNA damage checkpoint, chromosome morphology defects in late meiotic prophase I as well as impaired early embryogenesis and germline-specific changes in gene expression. Our results support the use of this high-throughput screening system to identify environmental chemicals inducing aneuploidy, and provide new insights into the effects of exposure to DBP, permethrin, and TCMTB on meiosis and germline function.

## Introduction

Man-made environmental chemicals such as phthalates, bisphenols, and pesticides, continue to increase in numbers, and some of them have been linked to reproductive problems [1–5]. However, rapidly identifying chemicals that impact reproductive health and understanding how they interfere with meiosis remains challenging. This is partly due to the fact that meiosis is not easily recapitulated in a tissue culture setting and that female mammalian meiosis can span from several months in mice to decades in humans. Failure to achieve accurate chromosome segregation during meiosis causes aneuploidy and can lead to infertility, stillbirths, miscarriages and birth defects [6,7]. Thus, high-throughput screens to assess the impact of environmental chemicals on reproductive health have been in high demand. *C*. *elegans* is a genetically and molecularly tractable model organism that provides many advantages for the study of meiosis and its use in high-throughput screens, including sharing a high degree of conservation of its genes and biochemical pathways with humans, carrying a well defined and characterized germline, a rapid life cycle (it develops from an egg into an adult in approximately 3 days at 20°C) and low maintenance costs [8–14].

Using chemotherapeutic agents and environmental compounds from the ToxCast Phase I library with comprehensive mammalian *in vivo* end point data (ToxRef database), we previously demonstrated that a *Pxol-1*::*gfp* transcriptional reporter strain in *C*. *elegans* can be used to identify chemicals inducing embryonic aneuploidy. Moreover, we showed that this approach is highly predictive of mammalian reproductive toxicity (balanced accuracy rate of 70%; this value corresponds to the average of sensitivity (correct identification of true positives) and specificity (correct identification of true negatives)) [15]. Here, for the first time, we successfully combined the use of this strain with sorting of live worms based on fluorescence intensity with a large object flow cytometry system, the COPAS Biosort (Union Biometrica), in a high-throughput screen. We screened a library of 46 chemicals consisting of pesticides, phthalates, and chemicals used in hydraulic fracturing and crude oil processing, selected based on their widespread presence in the environment and yet not well understood effects on the germline. Nineteen of these chemicals led to a GFP signal fold ratio over vehicle alone that was higher than the levels detected for bisphenol A (BPA) exposure, a widely used plasticizer and endocrine disruptor previously shown to affect meiosis in worms and mammals [1,16–20]. Three of these chemicals, dibutyl phthalate (DBP), permethrin and 2-(thiocyanomethylthio) benzothiazole (TCMTB), all high production volume chemicals, were evaluated further to validate the screening platform and gain insight into how they interfere with events in the germline.

DBP is a phthalate ester widely used as either a solvent or plasticizer. Therefore, it is found in a variety of items such as personal care products, plastic food wraps, the enteric-coatings of solid oral drug products, adhesives, and printing inks. DBP is highly prevalent in the environment and its estimated daily intake for the general population is 7–10 μg/kg/day [21–23]. Higher levels have been detected in human urine, follicular fluid, and serum in different occupationally exposed groups [21,24]. *In vitro* and animal studies have shown that DBP disrupts the reproductive system resulting in inhibition of ovarian antral follicle growth and viability, altered gene expression in ovaries, testicular malformation and dysfunction, inhibition of spermatogenesis, and altered androgen signaling in males [25–28]. In humans, a significant inverse relationship has been observed between levels of DBP metabolites (i.e. mBP) in prenatal urine and male anogenital distance [29]. Permethrin, a synthetic pyrethroid insecticide, is commonly used for crop protection and the treatment of head lice and scabies, since it is considered to have low toxicity compared to other insecticides [30]. Permethrin enters the body through skin, inhalation, and oral uptake. This chemical has been detected in adults and children [31–34] and its major metabolites, *cis*-permethrin and *trans-*permethrin, were detected at levels of 34 and 36 pg/ml in human cord serum, respectively [35]. Permethrin has been shown to alter gene expression and induce breaks in genes associated with leukemia and lymphoma in peripheral blood mononuclear cells exposed *in vitro* [36]. Finally, TCMTB is widely used as a wood preservative, an antimicrobial chemical for water systems, as a preservative for paper products, leather products, paints and wallpaper, and also as a pesticide in seed treatment for barley, cotton, corn, oats, rice and wheat [37]. While data on human intake is scarce, inhalation has been associated with testicular cancer in rodents (TCMTB risk assessment for the reregistration eligibility decision (RED) document, 2006). Importantly, the effects on meiosis from exposures to DBP, permethrin and TCMTB remain poorly understood.

Here we identified a set of chemical exposures affecting the germline and resulting in aneuploidy by using a high-throughput screening strategy in *C*. *elegans*. Subsequent analysis of three chemicals identified in this screen, DBP, permethrin, and TCMTB, provides new insights into their effects during meiosis and early embryogenesis. Exposure to these chemicals resulted in elevated DNA double-strand break (DSB) formation during meiosis and activation of a DNA damage checkpoint as indicated by elevated phosphorylated CHK-1 (pCHK-1) signal and p53/CEP-1-dependent germ cell apoptosis. Oocytes in diakinesis exhibited defects such as chromosome fragments suggestive of impaired meiotic DSB repair. Live cell imaging revealed chromosome segregation defects and spindle abnormalities during early embryogenesis. Finally, germline-specific expression for conserved DSB formation, repair and DNA damage checkpoint signaling genes is altered following the chemical exposures. These results show that this high-throughput screening platform can be successfully applied to rapidly and reliably identify chemicals affecting germline function and suggest that DBP, permethrin, and TCMTB interfere with maintenance of genomic integrity during meiosis and achieving accurate chromosome segregation.

## Results

### High-throughput screening of environmental chemicals encompassing phthalates, pesticides, and chemicals used in hydraulic fracturing and crude oil processing

**Fig 1:**
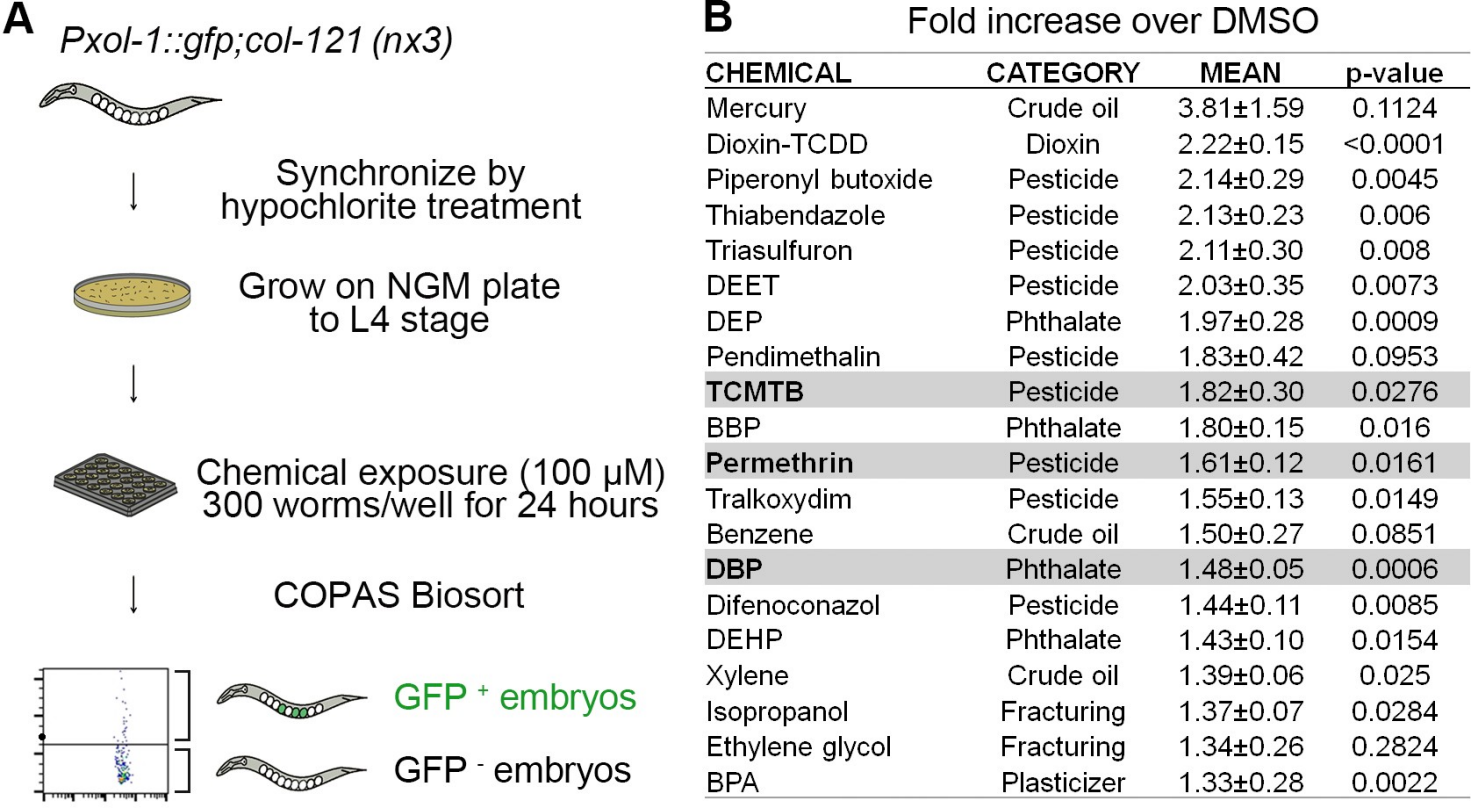
Flowchart for high-throughput screening strategy and readout. **(A)** Worms carrying a collagen gene mutation (*col-121(nx3)*) for increased cuticle permeability and a male-specific promoter driving GFP expression in embryos (*Pxol-1*::*gfp)*, were synchronized by hypochlorite treatment. Age-matched L1 stage animals were grown on plates (6,000 per 100 mm plate) until reaching the L4 stage. L4 stage animals were dispensed into 24-well plates (300 worms/well) with each well containing OP50 *E*. *coli* (OD600 = 24) in M9 buffer and a single chemical from our library (≤100 μM each) followed by a 24-hour incubation at 20°C. After thoroughly washed in M9, young adult animals were sorted based on fluorescence intensity with the COPAS Biosort. Adult worms with GFP^+^ embryos are detected as distinct from worms carrying GFP^-^ embryos and debris, which are below the threshold (black horizontal line). Threshold was determined by comparing levels of GFP^+^ embryos detected in two genetic mutants, *Pxol-1*::*gfp* and *Pxol-1*::*gfp;him-8*. More than 5,000 animals were assessed in biological triplicates for each chemical exposure. **(B)** Readouts obtained with the COPAS Biosort for the chemicals that showed higher fold increase in GFP^+^ embryos compared to DMSO than BPA, a known endocrine disruptor (mean ± SEM; *p*-values calculated by the paired two-tailed *t*-test, 95% C.I.). The three chemicals highlighted in gray, TCTMB, permethrin, and DBP, were assessed further for their effects on germline functions.

Our high-throughput screening platform takes advantage of two key features of *C*. *elegans*: their transparency and the rarity of males (X0<0.2% of offspring of self-fertilizing XX hermaphrodites; [38]), to identify chemical exposures that impair chromosome segregation in the germline and result in a high incidence of males. Increased X chromosome nondisjunction is detected using the reporter strain that has GFP expression controlled by a male-specific promoter (*Pxol-1*::GFP) [39,40], and carrying a collagen gene mutation (*col-121(nx3)*) that increases cuticle permeability without affecting the worm’s life cycle [41]. The *col-121* collagen gene mutation allows us to reduce the chemical concentrations to ≤100 μM, which are concentrations that circumvent lethality, are frequently used in chemical screens in *C*. *elegans*, and correlate well with mammalian reproductive endpoints ([42]; Fig 1A and Materials and Methods).

Briefly, we synchronized animals at the L1 larval stage by hypochlorite treatment and placed them on NGM plates with *E*. *coli* OP50 bacteria for food until the last larval stage (L4). Worms were exposed for 24 hours in 24-well plates starting at the L4 stage, when their gonads are fully formed, in liquid (M9 buffer) with OP50. Importantly, use of live bacteria is not detrimental resulting in low false-positive and–negative rates ([15,42,43]; S1A Fig). Gravid exposed mothers were then screened for increased incidence of GFP^+^ eggs (destined to become males) by comparison to vehicle alone (0.1% DMSO) using the COPAS Biosort (Union Biometrica), which allows for rapid sorting of live worms based on fluorescent intensities. We screened more than 5,000 animals in triplicate biological repeats for each chemical exposure. The 46 chemicals encompassed pesticides, phthalates, and chemicals used in hydraulic fracturing and crude oil processing (S1 Table). GFP positive signal above background from each chemical exposure was calculated as fold increase over DMSO (Fig 1B and S1 Table; also see Materials and Methods).

We identified nineteen chemicals showing higher fold increase over DMSO than BPA, an endocrine disruptor shown to affect meiosis leading to increased chromosome nondisjunction in worms and mammals (Fig 1B). Three of these are chemicals used in crude oil processing (mercury, benzene and xylene), with mercury scoring highest from among all chemicals tested. Reprotoxic effects have been previously reported for mercury, benzene and xylene [44,45]. Mercury bioaccumulates and can cause pathophysiological changes in the hypothalamus pituitary gland that may alter follicle-stimulating hormone (FSH) and preovulatory luteinizing hormone (LH) release thereby affecting reproductive function [44]. Women with occupational exposure to the hydrocarbons benzene and xylene have been shown to have reduced LH and mid-luteal phase pregnanediol 3-glucuronide (pd3G) as well as increased follicular-phase pd3G, which can cause reproductive abnormalities [45,46]. Nine of the chemicals were pesticides including thiabendazole, triasulfuron, and pendimethalin, which previous *in vivo* and *in vitro* studies have shown to be genotoxic and can result in mammalian germ cell aneuploidy [15,47–51]. Piperonyl butoxide, another pesticide in the list, has been shown to induce decreased female reproductive organ weight and histopathological changes in the ovary, uterus and vagina in rats likely due to its anti-estrogenic activity [52]. Four were phthalates (DEP, BBP, DBP, and DEHP), which are commonly added as solvents, additives and stabilizers to personal care products and medications and have adverse effects on reproductive and developmental health in humans (reviewed in [53]). Exposure to phthalates has also been shown to disturb sperm function [54,55], increase the percentage of pyriform sperm heads [56], increase DNA damage in sperm [57–60], impair mouse primordial follicle assembly *in vitro* [61] and reduce oocyte quality, embryonic developmental competency as well as alter expression of ovarian and pre-implantation embryonic genes in mice [62]. Finally, epidemiological studies suggest that exposure to isopropanol and ethylene glycol, chemicals used in hydraulic fracturing, may have a negative impact on human reproductive health [63]. The observed increase in X chromosome nondisjunction detected for all of these chemicals suggests effects in germline functions that will require further investigation. For further validation of this high-throughput screening strategy, we selected a phthalate, DBP, and two pesticides, TCMTB and permethrin. These were selected given that they elicited elevated levels of X chromosome nondisjunction and their reprotoxicity is less understood, allowing us to also gain more insight into their effects in germline function (see below).

### Exposures to DBP, permethrin, and TCMTB result in increased embryonic lethality and altered germline chromosome morphogenesis

**Fig 2:**
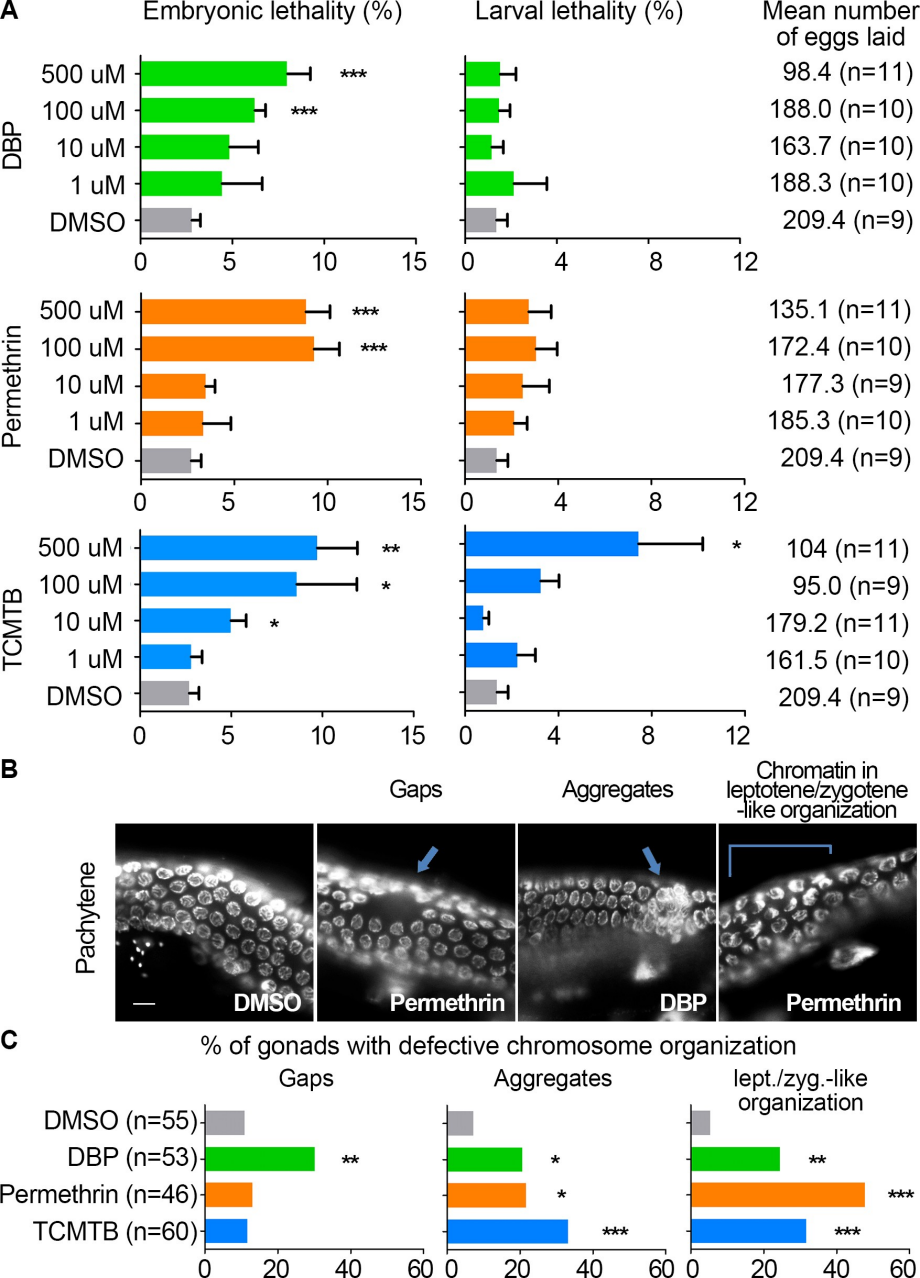
DBP, Permethrin, and TCMTB exposures result in increased embryonic lethality, sterility and defects in chromosomal organization in the germline. **(A)** Plate phenotypes for indicated chemical exposures. Embryonic lethality, larval lethality and the number of eggs laid (brood size) are shown for the indicated doses of exposures and compared to vehicle alone (0.1% DMSO). Error bars represent SEM. *P<0.05, **P<0.01, and ***P<0.001 by the two-tailed Mann-Whitney test, 95% C.I. **(B)** Images show pachytene nuclei in the germlines of whole, undissected worms fixed with Carnoy’s fixative and stained with DAPI that were exposed to vehicle control, 100 μM DBP, 100 μM permethrin, and 10 μM TCMTB. Chemical exposures lead to increased numbers of gonads with gaps (discontinuities or spaces lacking germ cell nuclei; arrow in second panel), aggregated nuclei (arrow, third panel) and DAPI-bright nuclei with chromosomes collapsed to one side (area indicated with a bracket, fourth panel) instead of dispersed throughout the nuclear periphery and organized in clear parallel tracks as seen in control at that stage. Scale bar, 5 μm. **(C)** Percentage of gonads exhibiting gaps, aggregates, or nuclei with chromatin in a leptotene/zygotene-like organization, in pachytene (n = number of gonads examined). * P<0.05, **P<0.01, and **P<0.001 by the two-sided Fisher’s exact test.

To assess whether DBP, permethrin and TCMTB affect chromosome segregation in general (including autosomes), and determine the dose of exposure for subsequent studies, we exposed worms to various concentrations of these chemicals (1, 10, 100 and 500 μM) for 24 hours starting at late L4, as in the high-throughput screen, and scored the number of eggs laid (brood size), embryonic lethality, and larval lethality. A decreased brood size and increased embryonic lethality can be due in part to defects during meiosis leading to errors in autosomal chromosome segregation and the consequent formation of aneuploid gametes in *C*. *elegans* [9,38,64]. We observed approximately a 50% reduction in the mean numbers of eggs laid on plates, which is indicative of increased sterility, for worms exposed to 500 μM DBP, 500 μM permethrin and both 100 μM and 500 μM TCMTB, compared to vehicle alone (Fig 2A). We also observed significantly increased embryonic lethality for exposures starting at 100 μM for DBP and permethrin (P<0.001; two-tailed Mann-Whitney test, C.I. 95%) and 10 μM for TCMTB (P<0.05) (Fig 2A). Furthermore, we observed higher larval lethality among the progeny of worms exposed to 500 μM TCMTB (P<0.05). These data further support and extend the results of our high-throughput screen suggesting that all three chemicals affect chromosome segregation in general and not limited to the X chromosome. Moreover, based on this analysis, doses of 100 μM for DBP and permethrin, and 10 μM for TCMTB, which result in embryonic lethality without significantly reducing the brood size or causing larval lethality, were used for all subsequent analysis.

To determine whether the increased chromosome nondisjunction is due in part to defects during meiosis, we examined DAPI-stained gonads from worms following exposures. In *C*. *elegans*, nuclei are positioned in a spatial and temporal gradient along the germline facilitating the identification of alterations in chromosome organization at specific meiotic stages [9]. We observed an increase in the number of gonads with gaps (areas with a reduced density of nuclei) in worms exposed to DBP compared to vehicle alone (30.2%, n = 53, and 10.9%, n = 55, respectively), as well as the presence of nuclei with DAPI-bright chromatin forming aggregates and nuclei with DAPI-bright and collapsed chromatin in a leptotene/zygotene-like organization at late pachytene in the gonads of worms exposed to all three chemicals compared to vehicle (aggregates: DBP: 20.8%, n = 53; permethrin: 21.7%, n = 46; TCMTB: 33.3%, n = 60; and DMSO: 7.3%, n = 55; leptotene/zygotene-like organization: DBP: 24.5%, n = 53; permethrin: 47.8%, n = 46; TCMTB: 31.7%, n = 60; and DMSO: 5.5%, n = 55) (Fig 2B and 2C). However, these defects are not due to overt impairments to early stages of meiotic progression or chromosome synapsis (S2A–S2E Fig). This is evidenced by normal localization of phosphorylated SUN-1 (SUN-1 S8), where SUN-1 corresponds to a conserved inner nuclear envelope protein with CHK-2- and PLK-2-dependent phosphorylation, with a signal appearing upon entrance into meiosis at the leptotene/zygotene stage and persisting on nuclei until mid-pachytene [65]. This is further supported by the normal localization of SYP-1, a structural component of the central region of the synaptonemal complex, observed associating with nuclei upon entrance into meiosis, forming full tracks between homologs at pachytene and starting to disassemble by late pachytene [66]. Taken together, these results show evidence of sterility, embryonic lethality and larval lethality as well as chromosome defects during pachytene after DBP, permethrin and TCMTB exposure, but these are not due to defects in meiotic progression or chromosome synapsis.

### DBP, permethrin, and TCMTB exposures result in increased germ cell apoptosis, elevated levels of meiotic DSBs and altered meiotic DSB repair

**Fig 3:**
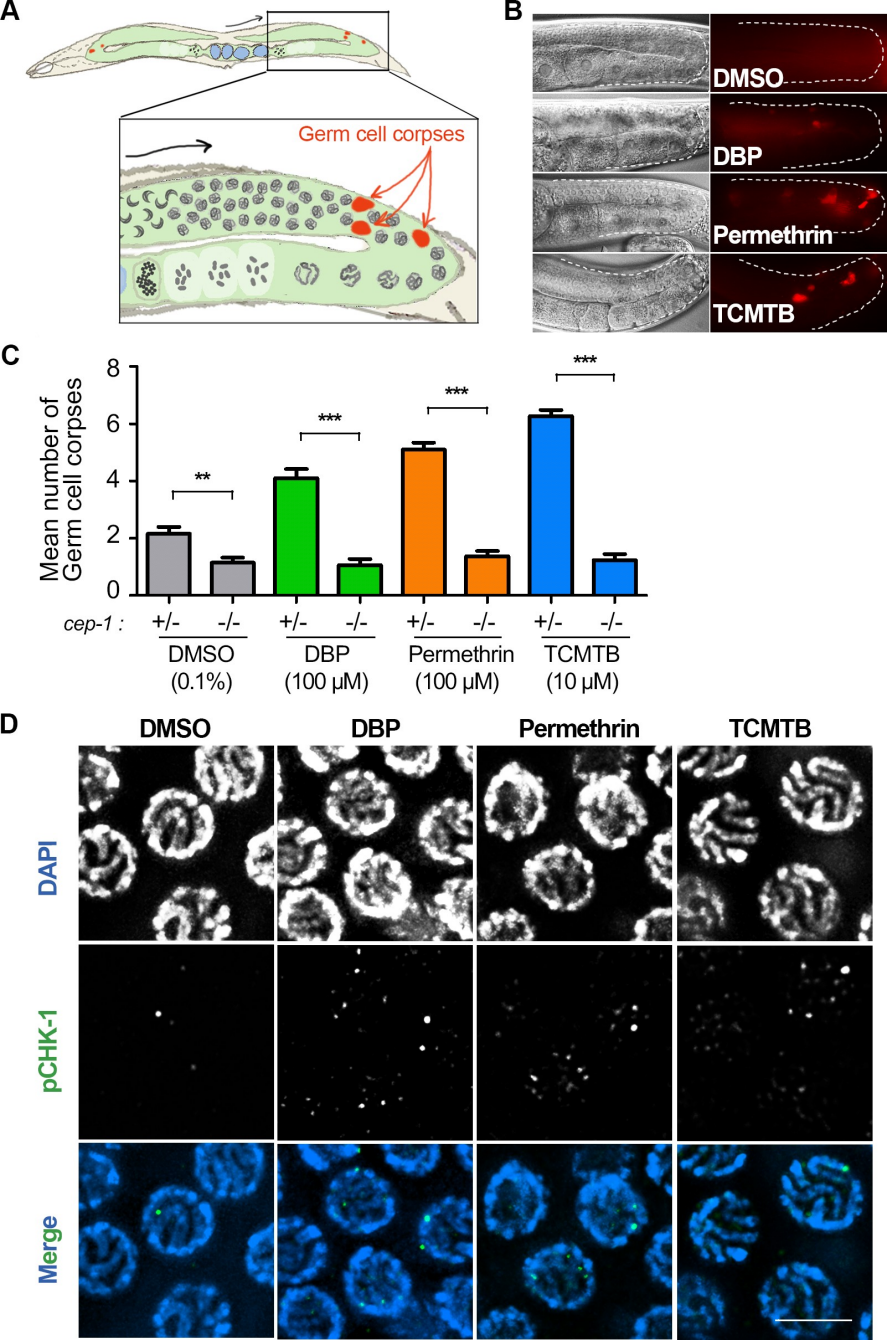
DBP, Permethrin, and TCMTB exposures lead to p53/CEP-1-dependent increased germ cell apoptosis and CHK-1 activation. **(A)** Schematic representation of *C*. *elegans* and the area where DNA damage checkpoint activation of germ cell apoptosis is detected in the germline. Inset represents zoom-in of one gonad arm. Black arrow indicates orientation of progression through meiosis while red arrows indicate germ cell corpses (red) observed at late pachytene near the gonad bend. **(B)** Chemical exposures caused a significant increase in the number of germ cell corpses observed at late pachytene compared to vehicle alone. Gonads are traced to facilitate visualization. On the left is the Nomarski optics view and to the right are the acridine orange stained germ cell corpses (red). **(C)** Graphical representation showing mean number of germ cell corpses detected for each indicated chemical. Levels of germ cell corpses were significantly reduced in a p53*/cep-1*-dependent manner. Note that basal level of toxicity for DMSO, as previously described [42,103], is also reduced in a *cep-1* mutant background. Analysis was done for three independent biological repeats. More than 30 gonads were scored for each chemical. Error bars represent SEM. **P<0.01, ***P<0.0001 by the two-tailed Mann-Whitney test, 95% C.I. **(D)** High-resolution images of mid to late pachytene nuclei from whole-mounted gonads immunostained for phospho CHK-1 (pCHK-1; green) and co-stained with DAPI (blue). Elevated levels of pCHK-1 were observed in chemical-treated worms compared to control. Scale bar, 5 μm.

Nuclei with DAPI-bright and collapsed chromatin at late pachytene have been previously correlated with germ cells undergoing apoptosis [67]. To determine whether the chemical exposures are causing increased germ cell apoptosis, we scored germline nuclei undergoing apoptosis (germ cell corpses, also referred to as apoptotic bodies) by acridine orange staining as in [39]. In wild type, animals exhibit less than three apoptotic bodies in late pachytene reflecting regular physiological apoptosis [64,68] (Fig 3A). We observed a significant 2- to 3-fold increase in the levels of germ cell corpses in late pachytene following all three chemical exposures compared to vehicle alone (Fig 3B and 3C). Moreover, the elevated germ cell apoptosis was observed in a dose-dependent manner, starting at 100 μM for DBP and permethrin, and 10 μM for TCMTB (S3 Fig). In *C*. *elegans*, physiological germ cell apoptosis does not depend on p53/CEP-1, which responds to genotoxic stress as a result of the activation of a DNA damage checkpoint [69]. Analysis of germ cell apoptosis levels in *cep-1;col-121* worms revealed the elevated apoptosis following all three chemical exposures was p53/CEP-1-dependent (Fig 3C). Activation of a DNA damage checkpoint was further supported by the increased signal detected in pachytene nuclei for a checkpoint kinase involved in DNA damage sensing, phosphorylated CHK-1 [70], following each chemical exposure (Fig 3D). Therefore, DBP, permethrin, and TCMTB exposures lead to activation of a DNA damage checkpoint resulting in increased p53/CEP-1-dependent germ cell apoptosis to clear affected nuclei.

**Fig 4:**
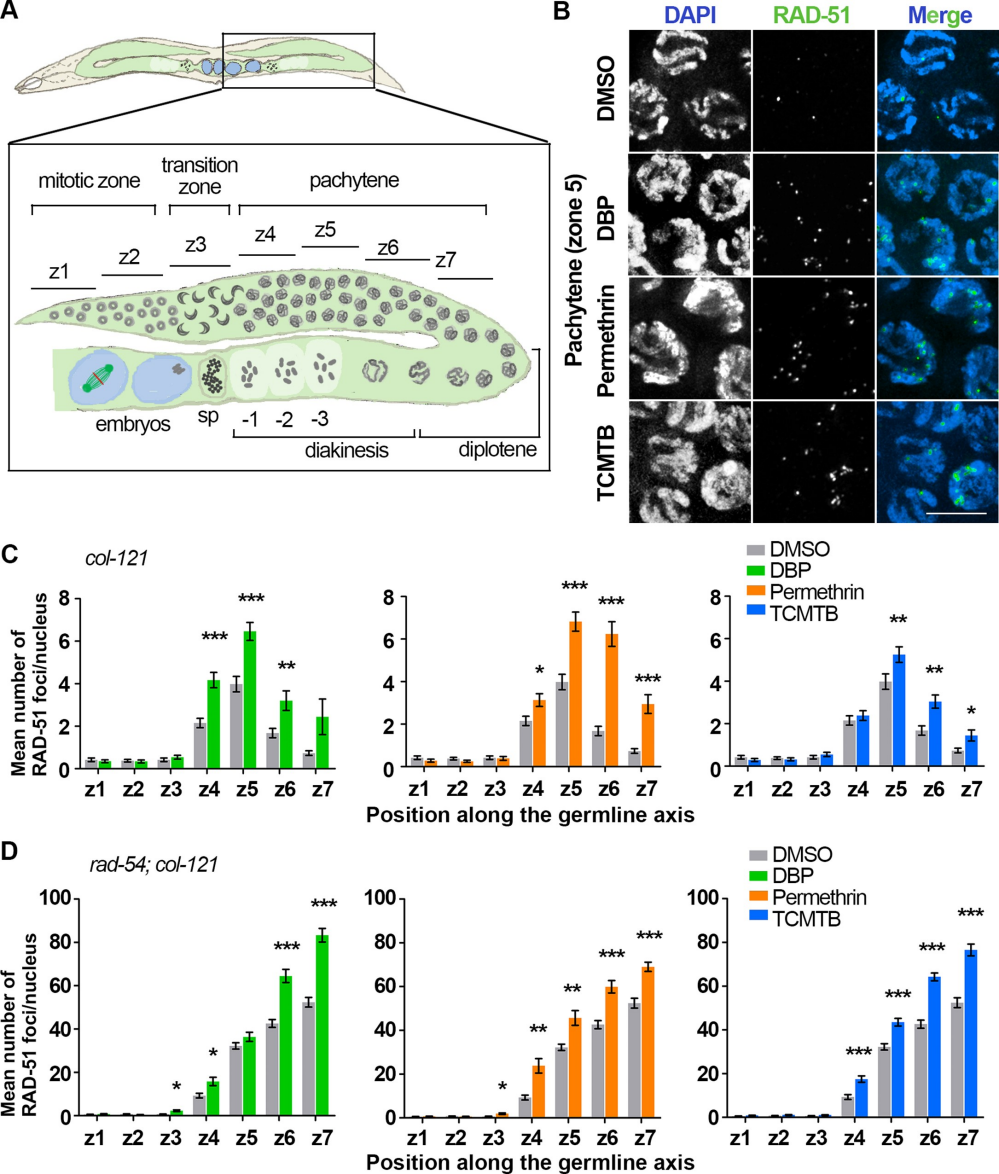
Chemical exposures result in increased DSB formation and impaired DSB repair. **(A)** Schematic representation of a *C*. *elegans* germline indicating the position of the equally sized zones (z1-z7) scored for RAD-51 foci. Nuclei in z1 and z2 are undergoing mitosis. They enter meiosis at z3 when they enter the transition zone, which corresponds to the leptotene/zygotene stages. Nuclei then proceed through pachytene (z4-z7), diplotene and diakinesis on their way into the uterus. sp: spermatheca. -1 indicates the oocyte closest to the spermatheca. **(B)** Representative images of pachytene nuclei (z5) immunostained for RAD-51 (green) and co-stained with DAPI (blue). Levels of RAD-51 foci in pachytene nuclei are elevated for each chemical exposure compared with control. Note that chromosomes in pachytene nuclei still exhibit a leptotene/zygotene-like organization in the cases of DBP and permethrin exposures. Scale bar, 5 μm. **(C)** Histograms show the mean number of RAD-51 foci scored per nucleus for each zone from *col-121* worms. Elevated levels of foci are observed persisting until late pachytene indicating a defect in DSB repair. >5 gonads from three independent biological repeats were scored for each indicated exposure. Error bars represent SEM. **(D)** Quantification of the mean number of RAD-51 foci scored per nucleus in *rad-54;col-121* worms. DSB levels were significantly higher upon the chemical exposures compared to vehicle alone. 3 gonads were scored for each chemical from two independent biological repeats. Error bars represent SEM. *P<0.05, **P<0.01, ***P<0.001 by the two-tailed Mann-Whitney test, 95% C.I.

The pachytene DNA damage checkpoint can be activated by the presence of unrepaired DSBs or aberrant recombination intermediates [64]. To examine this further, we quantified levels of RAD-51 foci as in [67]. RAD-51 binds to 3’ ssDNA ends at DSBs to promote strand invasion/exchange during DSB repair [71]. In vehicle-exposed gonads, like in wild type, low levels of RAD-51 foci were observed in nuclei at the premeiotic tip (zones 1–2) undergoing mitosis, as well as upon entrance into meiosis at transition zone where leptotene/zygotene stage nuclei are located (zone 3). Levels continued to rise throughout pachytene, peaking by mid-pachytene (zone 5), and then decreased by late pachytene (zone 7) as DSB repair progressed (Fig 4A–4C). In contrast, levels of RAD-51 foci were elevated specifically during meiosis for all three chemical exposures (Fig 4B and 4C). Levels of RAD-51 foci were indistinguishable from vehicle alone throughout the mitotic zone, but were higher than vehicle alone during pachytene. Moreover, analysis of *col-121* worms depleted of SPO-11, the protein required for meiotic DSB formation, further confirmed that the elevated levels of RAD-51 foci were SPO-11-dependent and therefore, meiotic-specific and not due to damage from the chemicals to the chromosomes (S4A–S4D Fig).

To determine whether the elevated levels of RAD-51 foci may be due in part to elevated DSB levels, we quantified RAD-51 foci in *rad-54*;*col-121* double mutants following chemical exposures. In a *rad-54* mutant, DSBs are formed and RAD-51 associates with DSB repair sites, but further repair is blocked essentially “trapping” DSB-bound RAD-51 and allowing for quantification of the total number of DSBs [72]. Levels of RAD-51 foci were significantly higher during meiosis for all three exposures compared to vehicle alone (Fig 4D). Taken together, these results suggest that exposures to DBP, permethrin and TCMTB result in elevated meiotic DSB levels and impaired DSB repair leading to activation of a DNA damage checkpoint and p53-dependent increased germ cell apoptosis.

### Chemical exposures lead to defects in late prophase I and first embryonic cell division

**Fig 5:**
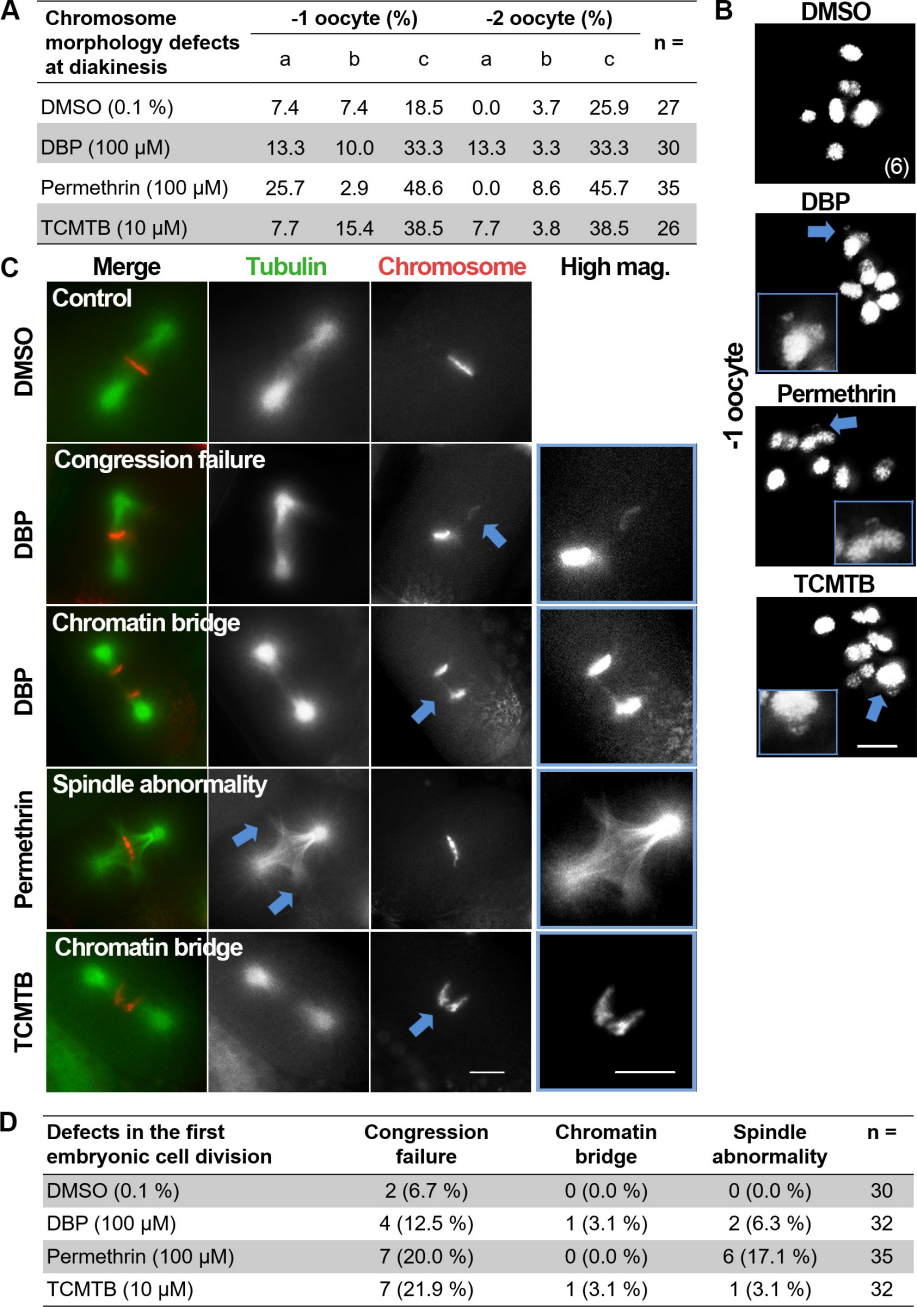
Chemical exposures lead to defects at diakinesis and the first embryonic cell division. **(A)** Quantification of the chromosome morphology defects observed in diakinesis (-1 and -2 oocytes). a: chromosome fragments, b: chromatin bridges, c: frayed chromosomes, n = total number of oocytes scored. **(B)** High resolution images of oocytes at diakinesis positioned right before the spermatheca (-1 oocyte). Six intact bivalents are observed in control. In contrast, frayed chromosomes, chromosome fragments and chromosome bridges (arrows and magnified in insets) are observed at higher levels in the germlines of chemical-exposed worms. Scale bar, 5 μm. **(C)** Representative images from time-lapse analysis of the first embryonic division in vehicle alone and DBP-, permethrin- and TCMTB-exposed H2B::mCherry; γ-tubulin::GFP; *col-121(nx3)* worms. A normal metaphase I configuration is shown for vehicle alone. Arrows and insets show examples of chromosomes that fail to align at the metaphase plate, chromatin bridges at the metaphase to anaphase transition and spindle abnormalities observed following exposures to all three chemicals. Scale bars, 5 μm. **(D)** Quantification of the time-lapse analysis of the first embryonic division. n = total number of embryos scored.

To determine whether exposures to DBP, permethrin and TCMTB might also result in defects at late prophase I, we examined chromosome morphology in oocytes at late diakinesis. In *C*. *elegans*, the six pairs of attached homologs (bivalents) are detected as six DAPI-stained bodies during diakinesis (Fig 5B). With high resolution microscopy we analyzed the -1 and -2 oocytes at diakinesis, which correspond to the two last oocytes proximal to the spermatheca (Fig 4A). We observed increased numbers of oocytes carrying chromosomes exhibiting a frayed morphology, chromatin bridges and chromosome fragments in germlines exposed to all three chemicals compared to vehicle alone (Fig 5A and 5B). This suggests that despite activation of p53-dependent apoptosis in late pachytene, some nuclei that failed to undergo normal DSB repair are progressing into late diakinesis.

To determine if DBP, permethrin and TCMTB exposures also impact early embryogenesis, we examined the first embryonic cell division by live imaging using transgenic worms carrying H2B::mCherry; γ-tubulin::GFP and the *col-121(nx3)* mutation. We detected the presence of lagging chromosomes (congression failure) and spindle abnormalities in the embryos examined from all three chemical exposures, and evidence of chromatin bridges in the metaphase to anaphase transition from DBP and TCMTB exposures compared to vehicle alone (Fig 5C and 5D). These results suggest that these chemical exposures affect meiosis as well as early embryogenesis, supporting the elevated chromosome nondisjunction detected by high-throughput screening following these treatments.

### Chemical exposures cause altered germline gene expression

**Fig 6:**
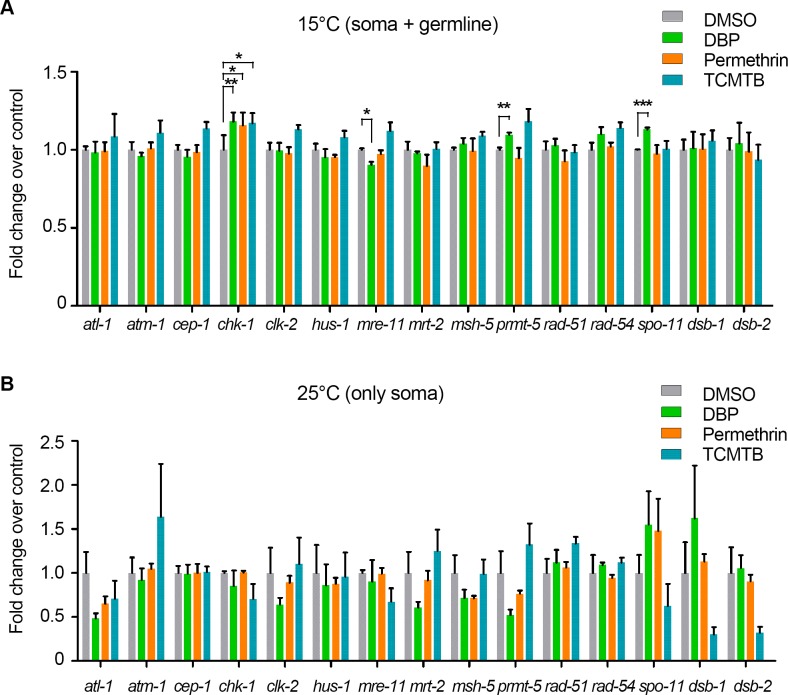
Germline-specific gene expression of DSB formation, repair and response genes is altered by chemical exposures. Expression levels of a panel of genes responsible for DSB formation, repair and DNA damage response were examined by quantitative RT-PCR in *glp-1(bn18)*;*col-121(nx3)* worms that grow without a germline when shifted from 15°C **(A)** to 25°C **(B)**. Thus, 15°C represents gene expression in both soma and germline, while 25°C represents expression only in the soma. The expression levels of 15 critical and conserved genes were measured from either three (15°C) or four (25°C) independent biological replicates (each performed with technical triplicates) and normalized to *gpd-1* (GAPDH). Y-axis indicates gene expression level change relative to vehicle alone. Error bars represent SEM. *P<0.05, **P<0.01, ***P<0.001 by the unpaired two-tailed *t*-test.

Given the effects on DSB formation, repair and DNA damage checkpoint activation observed during meiosis following all three chemical exposures, we next examined whether these defects arise from alterations in expression of DSB repair and DNA damage response genes. We examined mRNA levels by quantitative RT-PCR for 15 critical and conserved genes involved in these processes (Fig 6A and 6B). We used *glp-1*;*col-121* double mutants that develop as wild type at 15°C, but grow into adults lacking a germline when shifted to 25°C [73], to distinguish changes in gene expression occurring in the soma from those taking place in the germline. All three chemical exposures led to a significant increase in *chk-1* expression at 15°C (P<0.05), but not at 25°C (Fig 6A and 6B), indicating a germline-specific change in gene expression and correlating with the increased pCHK-1 foci we detected in the germline (Fig 3D). Moreover, DBP exposure resulted in germline-specific up regulation of *spo-11* and down regulation of *mre-11* (P<0.001 and P<0.05, respectively), which are factors involved in DSB formation and repair, as well as upregulation of *prmt-5* (P<0.01), involved in regulation of DNA damage-induced apoptosis [74,75]. Taken together, these results suggest that germline-specific alterations in the expression of genes involved in DSB formation, repair and response may contribute in part to the defects in maintaining genomic integrity and achieving accurate chromosome segregation observed in the germline following these chemical exposures.

## Discussion

We showed that a high-throughput screening strategy can be successfully applied to identify environmental chemicals causing aneuploidy using the nematode *C*. *elegans*. Due to the increasing number of chemicals being introduced into the environment and their broad uses, strategies for rapidly assessing toxicity, in a manner predictive of their effects on human health, are in high demand. *C*. *elegans* is a metazoan system that offers various advantages for this type of analysis including low maintenance costs, a rapid life cycle and a high degree of conservation of its genes and biochemical pathways with humans [9,76–80]. This nematode is also being successfully used in high-throughput screens of compounds and genes impacting pathways related to human disease [11,13,81–83]. *C*. *elegans* is also an ideal model system specifically for studying the effects of chemical exposures on the germline since genes and pathways involved in regulating key processes such as germ stem cell renewal and differentiation, meiosis, ovulation and embryogenesis are conserved between *C*. *elegans* and humans. Moreover, its germline is well characterized and amenable to studies using genetic, biochemical, and molecular biology tools combined with powerful cytological approaches [8,12,14]. Our high-throughput screen identified several different classes of chemicals that are leading to increased chromosome nondisjunction and follow up studies will further explore how they are affecting the germline. Along those lines, here we also provided new insights into the effects of DBP, permethrin and TCMTB on germline functions.

**Fig 7:**
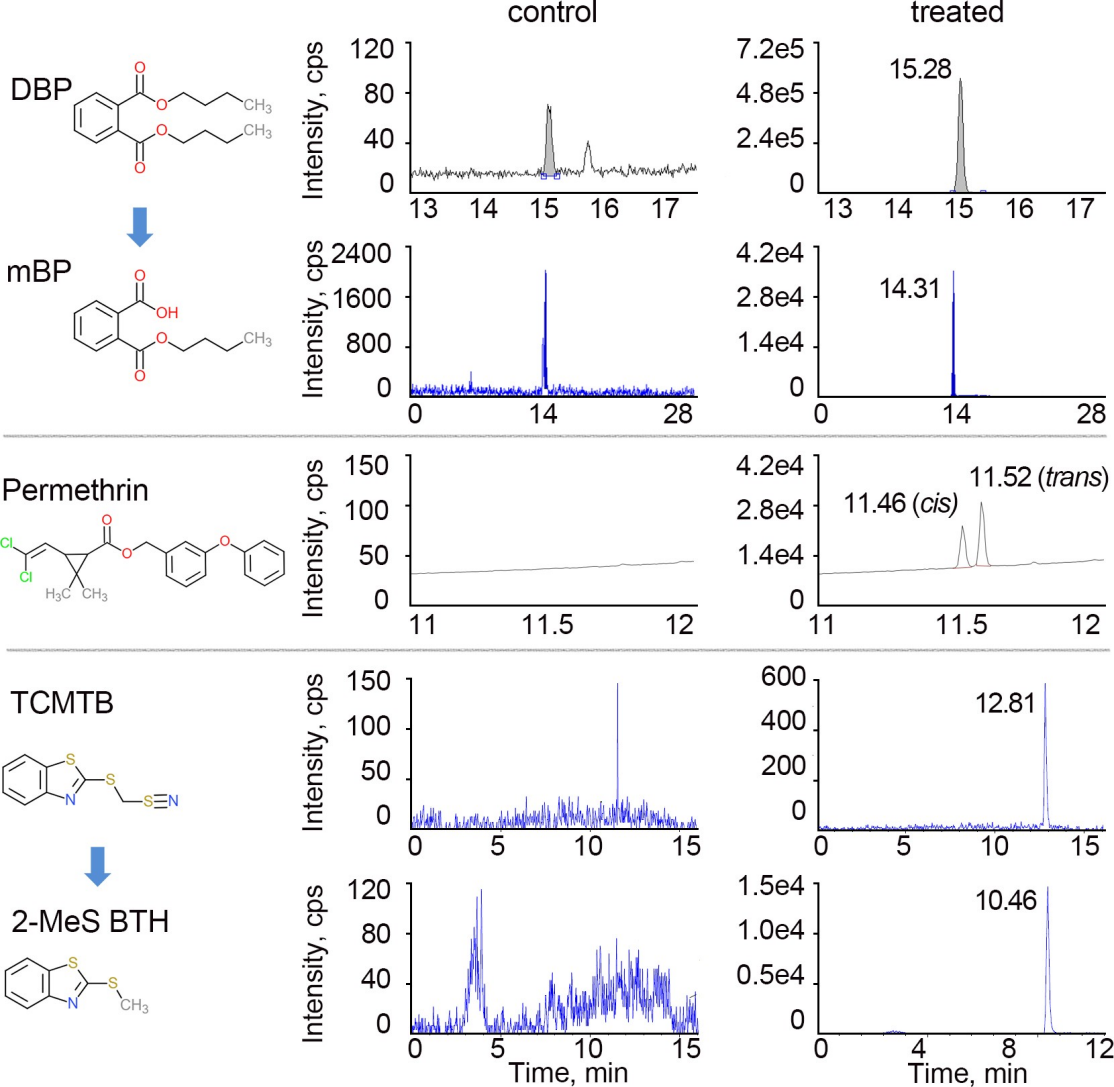
Structures and representative LC-MS/MS and GC-MS chromatograms for DBP, permethrin, TCMTB and their metabolites. Chromatograms on the left show results from the analysis of the indicated chemicals and metabolites in worms exposed to vehicle alone (DMSO; control) and chromatograms on the right show results from worms exposed to DBP, permethrin, and TCMTB (treated). These chemicals were unambiguously identified and quantified using their respective internal standards. Y-axis represents relative abundance of signal intensity and X-axis represents retention time in minutes. The detected concentrations following chemical treatments (right) were consistently greater than in control (note differences in Y-axis). Note that the peak at 12.81 minutes does not correspond to TCMTB (expected peak for TCMTB is at 8.76), suggesting that TCMTB is very rapidly metabolized in the worm.

DBP is metabolized by esterases to form mono-n-butyl phthalate (mBP) once it enters the body, while permethrin undergoes hydrolysis and oxidation in the liver by carboxylesterases and cytochrome P450 to conjugated and unconjugated *cis/trans* 3-(2,2-dichlorovinyl)-2,2-dimethylcyclopropane carboxylic acid (CVA) with their plasma levels reaching peak values in 5–7 hours [84–86], and TCMTB is converted into cyanide, 2-mercaptobenzothiazole (2-MBT) by cytochrome P450 in the liver [87]. In our study, we exposed worms for 24 hours to 100 μM or 27.8 μg/ml of DBP, which our dose-response studies showed impaired chromosome segregation with low overall toxicity, as determined by both growth and behavior of the worms. Measurement of the internal concentration from whole worm extracts by isotopic dilution mass spectrometric analysis revealed internal levels of 8.9 μg/ml for DBP, and 2.2 μg/ml for mBP, confirming that this chemical reaches internal circulation (Fig 7). A study of a small group of women undergoing IVF in the USA detected a median value of 1.46 ng/ml of mBP in follicular fluid [88] while a larger study of 110 women undergoing IVF in China detected median values for mBP of 2.05 ng/ml in follicular fluid and 102.30 ng/ml in urine and maximum values for DBP of 415 ng/ml in follicular fluid and 2.32 μg/ml in urine [89]. Studies showing the relationship between prenatal phthalate exposure and anogenital distance (AGD) as an outcome of reproductive toxicity detected geometric means for mBP of 67.62 ng/ml in urine from 196 women in Sweden and 15.04 ng/ml in urine from 380 women in the USA [29,90]. This suggests that the levels which resulted in germline defects in *C*. *elegans* are within the range relevant to human exposures. In this study, worms were exposed for 24 hours to external doses of 100 μM permethrin and 10 μM TCMTB, corresponding to 39.13 μg/ml and 2.38 μg/ml, respectively. Measurement of the internal concentrations from whole worm extracts revealed internal levels of 5.6 μg/ml for *cis-* and *trans-*permethrin combined, and 0.5 μg/ml for 2-methylthio benzothiazole (2-MeS BTH), showing that these chemicals also reach internal circulation (Fig 7). TCMTB is apparently metabolized very rapidly in worms (akin to mammals [87]) and we were unable to detect it in our extracts. We also did not detect either benzothiazole (BTH) or 2-hydroxy benzothiazole (2-OH BTH) metabolites. Unfortunately, data on the concentrations of these metabolites in non-blood tissue or organs in either mammalian models or humans is scarce to nonexistent, thus limiting comparisons regarding exposure levels. However, the internal levels detected for DBP and mBP, coupled with the effects on germline functions detected by our high-throughput screen and follow up studies for all three chemicals, suggest that *C*. *elegans* offers the necessary sensitivity to detect effects at environmentally relevant doses of exposure.

Here we showed that all three chemicals resulted in elevated meiotic DSB levels, impaired DSB repair, activation of p53-dependent germ cell apoptosis and elevated phosphorylated CHK-1 signal during late pachytene, along with chromosomal abnormalities in oocytes at late diakinesis and impaired chromosome segregation during early embryogenesis. *In vitro* studies of the effects of DBP exposure on Sertoli cell culture showed increased apoptosis stemming from inhibiting the PI3K/AKT and mTOR pathways which promote the proliferation and survival of sperm and the maintenance of testicular homeostasis [26]. Antral follicles isolated from female mice and exposed to DBP exhibited increased expression of the cyclin-dependent kinase inhibitors *Cdkn1a* and *Cdkn2a* and pro-apoptotic factors *Bax* and *Bid* along with down regulation of cyclin *Ccnd2* resulting in growth inhibition and follicular death [28]. Permethrin has been shown to cause DNA damage in mitotic cells where exposure of peripheral blood mononuclear cells (PBMCs) induced breaks in the *KMT2A* and *IGH* genes which can be driver mutations for lymphoma and leukemia along with increased aneuploidy [36]. TCMTB has been reported as exhibiting relatively low toxicity upon either oral or dermal uptake since it is rapidly metabolized into 2-MBT in the body and excreted through the urinary tract, however it has been considered highly toxic via the inhalation route and resulted in increased incidence of testicular interstitial cell adenomas in male rats [87], (TCMTB risk assessment for the reregistration eligibility decision (RED) document, 2006).

To our knowledge, the germline-specific upregulation of *chk-1* following exposures to DBP, permethrin and TCMTB, and the downregulation of *mre-11* along with the upregulation of *spo-11* and *prmt-5* following DBP exposure, have not been previously reported. This altered gene expression profile is congruent with the elevated meiotic DSB formation, altered meiotic DSB repair and activation of a DNA damage checkpoint observed during meiosis. SPO-11 is the topoisomerase-like conserved protein that catalyzes meiotic DSBs so that the elevated levels of DSB formation observed in DBP exposed worms may be due in part to deregulation of *spo-11* expression. *prmt-5* encodes for the ortholog of human PRMT5, a protein arginine methyltransferase involved in the post-translational modification of a variety of proteins including histones and G protein-coupled receptors, thereby regulating transcription and signaling [91]. Interestingly, PRMT5 has been proposed to regulate the target gene specificity of p53 in mammals [74] and to negatively regulate apoptotic signaling in response to DNA damage in *C*. *elegans* by repressing p53/CEP-1 transcriptional activity through downregulation of *cbp-1*/p300, which encodes for a cofactor of CEP-1 [75]. Alternatively, upregulation of *prmt-5* may result in alterations in transcription or chromatin accessibility, for example via its role in histone regulation, which in turn could also contribute to elevated DSB formation and/or altered repair. Finally, *mre-11* encodes for a member of the MRX/N (Mre11, Rad50, Xrs2/Nbs1) complex required for meiotic DSB formation and resection [92–97]. The elevated levels of DSBs and RAD-51 foci detected following DBP exposure suggest that downregulation of *mre-11* may not be interfering with DSB formation and/or end resection in this case. Finally, although all three chemical exposures led to elevated germline-specific *chk-1* gene expression, additional studies will be required to determine how permethrin and TCMTB affect DSB formation and repair during meiosis.

Taken together, our results demonstrate that a high-throughput screening platform can be used in *C*. *elega*ns to successfully identify environmental chemicals affecting the germline. Moreover, our findings revealed the effects of DBP, permethrin, and TCMTB exposures on the germline and potential mechanisms by which DBP affects germline functions, broadening our understanding of the potential effects of environmental toxicants on human reproductive health.

## Materials and methods

### *C*. *elegans* strains

*C*. *elegans* strains were cultured at 20°C under standard conditions as described in [98]. The following mutations and chromosome rearrangements were used in this study: LGI, *cep-1(lg12501)*, *rad-54(ok615)*; LGIII, *glp-1(bn18)*; LGIV, *col-121(nx3)*, *him-8(e1489)*; LGV, *yIs34*[*Pxol-1*::*GFP*, *rol-6*].

### Calibration of COPAS Biosort parameters and gating of adult population

To ensure the GFP signals detected in the COPAS Biosort stemmed only from gravid worms and not debris, we first synchronized worms at the L1 stage by hypochlorite treatment as in [99] and sorted either *Pxol-1*::*gfp; col-121* or *Pxol-1*::*gfp; col-121; him-8(e1489)* worms at different developmental stages (L1 through young adults). Presence of the *him-8* mutation results in a high incidence of male progeny (36.7%; [38]) due to increased X chromosome nondisjunction. The use of both strains allowed us to exclusively gate adult worms and establish the threshold for worms carrying GFP+ embryos above background. Thus, only an adult population from age-matched animals underwent screening (S1B–S1D Fig).

### Chemical exposure and screening with the COPAS Biosort

Age-matched embryos were obtained from gravid worms following sodium hypochloride treatment and subjected to overnight starvation [99]. Thoroughly washed age-matched L1-stage worms were grown on regular NGM plates up to the L4 stage (5,000 to 6,000 L1 worms were grown on each 100 mm plate). Between twenty to thirty thousand L4-stage animals were resuspended in M9 buffer with freshly cultured OP50 bacteria (OD600 = 24). 300 worms in 250 μl of M9 with OP50 bacteria were dispensed, along with each chemical, into individual wells in 24-well plates. All chemicals, except TCDD, were purchased from Sigma Aldrich (St. Louis, MO) and dissolved in DMSO at 0.1 M except for polyacrylamide and hydroxyethyl cellulose, which were dissolved in water respectively at 20 mg/ml and 33 mg/ml for solubility reasons. TCDD was purchased from AccuStandard (New Haven, CT). Final DMSO and chemical concentrations were 0.1% and 100 μM, respectively, except for dicofol, mancozeb, parathion-methyl, phosalone, pyridaben, TCMTB, arsenic oxide, and mercury, which were further diluted 10-fold to circumvent lethality. Chlorpyrifos-methyl and TCDD were used at 1 μM and 100 nM, respectively, for the same reason. After 24 hours, the exposed worms were transferred to 1.5 ml tubes, washed five times with M9, and utilized for subsequent experiments and analysis through the COPAS Biosort (Union Biometrica, Holliston, MA). Time-of-flight (Tof) and GFP peak height were used as reading parameters in the COPAS Biosort. Three independent biological repeats, encompassing a total of more than 5,000 worms for each chemical exposure, were run through the COPAS Biosort. Fold-increase GFP^+^ signal over DMSO was calculated for each biological repeat and then an average of the fold increase was calculated (Fig 1B).

### Scoring embryonic lethality, larval lethality and sterility

Age-matched worms were exposed to either vehicle alone (DMSO) or each chemical in liquid for 24 hours as described above. After 24 hours, the exposed worms were washed five times with M9, and transferred to regular NGM plates to score their embryonic lethality, larval lethality and sterility. Worms were moved every 24 hours to new NGM plates (this was done for three consecutive days). The total number of fertilized eggs laid, hatched, and the number of progeny that reached adulthood were scored.

### Germ cell apoptosis

Germ cell corpses were scored as in [39], utilizing a Leica DM5000B fluorescence microscope. The germlines of more than 30 worms from at least two independent biological repeats were scored for each chemical exposure. Statistical comparisons between groups were performed using the two-tailed Mann-Whitney test, 95% C.I.

### Immunofluorescence microscopy

Whole mount preparation of dissected gonads and immunostainings were performed as in [67]. Primary antibodies were used at the following dilutions: goat α-SYP-1(1:3,000; [100]), rabbit α-pCHK-1(1:100; Santa Cruz), guinea pig α-pSUN-1 Ser8-pi (1:700; [65]), and rabbit α-RAD-51 (1:10,000; Novus Biological (SDI)). The following secondary antibodies from Jackson ImmunoResearch Laboratories (West Grove, PA) were used at a 1:200 dilution: α-rabbit Cy3, and at a 1:500 dilution: α-goat Alexa 647, α-rabbit Alexa 488, and α-guinea pig Alexa 488. Vectashield from Vector Laboratories (Burlingame, CA) was used as a mounting media and anti-fading agent.

Immunofluorescence images were collected at 0.2 μm intervals with an IX-70 microscope (Olympus, Waltham, MA) and a cooled CCD camera (CH350; Roper Scientific) controlled by the Delta Vision system (Applied Precision, Pittsburgh, PA). Images were subjected to deconvolution by using the SoftWoRx 3.3.6 software (Applied Precision).

### Time course analysis for RAD-51 foci

Quantitative analysis of RAD-51 foci for all seven zones composing the germline was performed as in [67]. The average number of nuclei scored per zone (n) from 3 to 6 gonads for each chemical-treated group was as follows, ± standard deviation: For the *col-121* line: zone 1 (n = 80.3±4.3), zone 2 (n = 93.5±7.4), zone 3 (n = 97.0±9.3), zone 4 (n = 86.3±6.6), zone 5 (n = 59.3±8.5), zone 6 (n = 56.5±1.7), zone 7 (n = 57.8±10.7). For the *rad-54;col-121* line: zone 1 (n = 56.5±5.8), zone 2 (n = 67.0±6.2), zone 3 (n = 59.8±5.2), zone 4 (n = 46.0±6.6), zone 5 (n = 39.8±7.8), zone 6 (n = 31.5±5.4), zone 7 (n = 26.3±2.5). Statistical comparisons were performed using the two-tailed Mann-Whitney test, 95% C.I.

### RNA interference

Feeding RNAi experiments were performed at 20°C in *col-121* mutants as described in [101] with the following modifications: three L4-stage animals were placed on each RNAi plate and F3 generation L4-stage worms were used for chemical exposures at 25°C. HT115 bacteria expressing empty pL4440 vector was used as the control RNAi.

Strong RNAi knockdown of *spo-11* results in oocytes with 12 DAPI-stained bodies due to the lack of meiotic DSBs and subsequent crossovers leading to 6 unattached pairs of homologs. We verified that 100% (n>22) of the oocytes for each chemical exposure exhibited 12 DAPI-stained bodies. The effectiveness of RNAi was also confirmed by RT-PCR from at least four individual worms subjected to RNAi. Expression of *gpd-1* (GAPDH) transcript was used as a control.

### Quantitative RT-PCR analysis

Three samples of 20 (15°C) to 30 animals (25°C) each were collected in 100 μl of Trizol (Invitrogen) and RNA was extracted according to the manufacturer’s instructions. The extracted RNA was subjected to reverse transcription using iScript (Biorad) and quantitative real time PCR was performed using SsoFast EvaGreen supermix (Biorad) according to the manufacturer’s instructions. Each sample was run in triplicate. Cq numbers were normalized to *gpd-1*, then the normalized values from DBP, permethrin, and TCMTB treated samples were statistically compared with the normalized values from vehicle (DMSO) treated samples. Bars in graphs show mean values normalized to DMSO ± SEM. Statistical comparisons were performed using the unpaired two tailed *t*-test, 95% C.I.

### Live imaging

Strain CV639 (H2B::mCherry; γ-tubulin::GFP;*col-121(nx3)*) was used for live imaging and worms were immobilized with 0.01% levamisole on 3% agarose pads. Images were captured with a 60X objective every 10 seconds on an IX-70 microscope (Olympus, Waltham, MA) and a cooled CCD camera (CH350; Roper Scientific) controlled by the DeltaVision system (Applied Precision, Pittsburgh, PA).

### Preparation of worm lysates for mass spectrometric analysis

After exposure to each chemical as described above, worms were washed 10 times in M9 buffer and frozen with minimal M9 in liquid nitrogen. The worm pellet was resuspended in lysis buffer [0.5 M sucrose, 25 mM HEPES (pH7.6), 5 mM EDTA, 0.5% CHAPS, 0.5% DOC (Deoxychloric acid)]. Samples were then sonicated at 4°C for 10 cycles (1 minute on and 1 minute off per cycle) with a Bioruptor Plus 300 (Diagenode, Belgium).

### Mass spectrometric chemical analysis

DBP was extracted from worms/lysates using hexane and analyzed using gas chromatography-mass spectrometry (GC-MS). A Thermo trace 1310 GC and a HP-5MS capillary column (30 m×0.25 mm×0.25 μm) interfaced with ISQ single quadrupole mass spectrometer (Waltham, MA, USA) was used for the analysis. Procedural blanks and matrix spikes were included for quality control purposes along with the analysis of control/vehicle and treated *C*. *elegans*. The trace level DBP found in the procedural blank was subtracted from sample values to report the final concentration. The matrix spike recovery was 91.6%.

mBP (the metabolite of DBP) in worms was analyzed using a method described for urine earlier, with some modifications [102]. Briefly, the worm lysates were enzymatically (β-glucuronidase) deconjugated followed by extraction using a solid-phase extraction (SPE) method with a solvent mixture of acetonitrile and ethyl acetate. An API 4500 electrospray QTRAP mass spectrometer (ESI-MS/MS; Applied Biosystems, AB Sciex, Framingham, MA, USA) operated in the negative mode of ionization interfaced with an Agilent 1260 HPLC (Agilent Technologies Inc., Santa Clara, CA) was used for the analysis of mBP. Quantification of mBP was achieved by an isotopic dilution method.

Permethrin was extracted from worm lysates using a similar protocol to that applied for DBP analysis. A 1:2 ratio of hexane and dichloromethane solvent mixture was used for the extraction and analysis was performed using an Agilent single quadrupole GC-MS under electron ionization mode. The matrix spike recoveries for both *cis-* and *trans-*permethrin were 112% and 101%, respectively.

For TCMTB analysis, worm lysate was spiked with 40 ng of D4-Benzothiazole (internal standard) and maintained at room temperature for equilibration (15 min). Methanol and acetone (1:1 ratio) solvent mixture was used for the extraction of target chemicals. The extracts were centrifuged and filtered through 0.2 μM nylon membrane filters and transferred into HPLC amber vials. A Shimadzu Prominence Modular HPLC system (LC-20 AD UFLC; Shimadzu Corporation, Kyoto, Japan) equipped with an Agilent Zorbax SB-Aq column (2.1 mm X 150 mm, 3.5 mm; Santa Clara, CA, USA) serially connected with an AB SCIEX 3200 triple quadrupole mass spectrometer was used for the identification and quantification of TCMTB and its metabolites (Benzothiazole (BTH), 2-methylthio benzothiazole (2-MeS BTH) and 2-hydroxy benzothiazole (2-OH BTH)) under the positive electrospray ionization mode. Although we screened for three major possible metabolites of TCMTB, we could detect only 2-MeS BTH in the treated worms. The gradient mobile phase (A: acetonitrile and B: water that contains 0.1% formic acid) was eluted at a flow rate of 300 μL/min for the effective separation of target chemicals.

## Supporting information

S1 Fig Calibration and validation of the automated fluorescence reading platform. **(A)** Mean number of germ cell corpses detected for DMSO (0.1%) and DBP (100 μM) exposed worms grown in the presence of either live or heat inactivated (dead) OP50 *E*. *coli*. There were no differences detected within DMSO or DBP groups (NS), demonstrating that the effects were not due to the live bacteria (i.e. their response to the chemical stressor) in the media. Analysis was done for two independent biological repeats. More than 30 gonads were scored for each chemical. Error bars represent SEM. ***P<0.0001 by the two-tailed Mann-Whitney test, 95% C.I. **(B)** L2, L3, L4 and adult populations were sorted through the COPAS Biosort and used to draw a gate capturing only the adult population. The population in this gate was measured for GFP fluorescence intensity. ToF, time-of-flight. **(C)** Two genetic mutants, *Pxol-1*::*gfp* and *Pxol-1*::*gfp;him-8*, were used to define the threshold for GFP^+^ embryos, GFP^-^ embryos and debris. Reading parameters used were ToF for the x-axis and GFP peak height for the y-axis. **(D)** The induction of X chromosome nondisjunction can be visualized by fluorescence microscopy. GFP^+^ embryos are visible within the chemical treated worm’s uterus (arrows). Two chemicals that elicit increased chromosome nondisjunction, nocodazole, a microtubule disruptor, and triflumizole, a pesticide [15], were used as positive controls. Asterisks indicate gut autofluorescence.
(TIF)

S2 Fig Chemical exposures do not affect meiotic progression. **(A-D)** Low magnification images of whole mounted gonads immunostained for SYP-1 (red), a central region component of the synaptonemal complex (SC), and phosphorylated SUN-1 (SUN-1 S8pi; green), a marker for progression of early prophase I events. All three chemical exposures exhibit normal timing of SC assembly and disassembly and normal duration of SUN-1 S8 signal compared to vehicle. Dissected gonads are oriented from left to right as indicated by the white arrows. Blue vertical bar indicates entrance into meiosis. Gonads are outlined to facilitate visualization. Scale bars, 10 μm. **(E)** High magnification images of mid pachytene nuclei from whole-mounted gonads immunostained for SYP-1 (red) and co-stained with DAPI (blue). Continuous full tracks of SYP-1 were observed between homologs in mid pachytene. Scale bar, 5 μm.
(TIF)

S3 Fig Chemical exposures induce increased germ cell apoptosis in a dose-dependent manner. Chemical exposures caused a significant increase in the number of germ cell corpses observed at late pachytene compared to vehicle alone starting at 100 μM for DBP and permethrin and at 10 μM for TCMTB. Germ cell corpses from more than 30 gonads were scored for each chemical exposure. Error bars represent SEM. **P<0.01, ***P<0.0001 by the two-tailed Mann-Whitney test, 95% C.I.
(TIF)

S4 Fig Elevated meiotic DSB levels observed following chemical exposures are SPO-11-dependent. **(A, B)** Representative images of pachytene nuclei (z5) immunostained for RAD-51 (red), co-stained with DAPI (blue) and high magnification images of oocytes at diakinesis positioned right before the spermatheca (-1 oocyte) in *col-121* control (empty vector) RNAi (A) or *col-121 spo-11(RNAi)* (B). Six bivalents are observed in 100% (n = DMSO: 11, DBP: 10, permethrin: 25, and TCMTB: 18) of the oocytes scored in control RNAi. In contrast, 12 univalents are observed in 100% (n = DMSO: 28, DBP: 29, permethrin: 25, and TCMTB: 22) of the -1 oocytes upon SPO-11 depletion indicating that RNAi depletion worked effectively. While elevated levels of RAD-51 foci are observed in pachytene nuclei for all indicated chemical exposures compared with control, scarcely any RAD-51 foci are observed in pachytene nuclei for either the chemical exposures or vehicle alone when SPO-11 is depleted. Scale bar, 5 μm. **(C)** Histograms show the mean number of RAD-51 foci scored per nucleus for each zone from *col-121* control(RNAi) (C) and *col-121 spo-11(RNAi)* worms (D). Three gonads from two independent biological repeats were scored for each indicated exposure. Error bars represent SEM. *P<0.05, **P<0.01, ***P<0.001 by the two-tailed Mann-Whitney test, 95% C.I. **(D)** RT-PCR of *col-121 spo-11(RNAi)* compared to *col-121 control(RNAi)* (empty vector). Each lane corresponds to a single worm lysate and indicates the effective depletion of *spo-11* by RNAi (shown are the single worm lysates from vehicle alone). *gpd-1* expression was used as a loading control.
(TIF)

S1 Table Readouts from high-throughput screening of the environmental chemicals with the COPAS Biosort. Categories indicate the class or use of the chemicals tested (pesticide, phthalate, crude oil processing and hydraulic fracturing; exceptions are TCDD and BPA listed as dioxin and plasticizer, respectively). Concentrations assessed for each chemical in the high-throughput screen are indicated (all chemicals were diluted in DMSO). Chemicals are ranked based on the fold increase in GFP^+^ embryos detected compared with DMSO-treated (vehicle alone) worms. A minimum of 5,000 worms were screened for each chemical.
(TIF)

S2 Table Raw data set. Raw data for plate phenotyping, apoptotic nuclei count, RAD-51 foci count, and qRT-PCR analysis.
(XLSX)
