# Supplementary figures and images for: Assessing effects of germline exposure to environmental toxicants by high-throughput screening in C. elegans
Source: PLoS Genet. 2019 Feb 14;15(2):e1007975. doi: 10.1371/journal.pgen.1007975 (PMC6375566; doi:10.1371/journal.pgen.1007975)

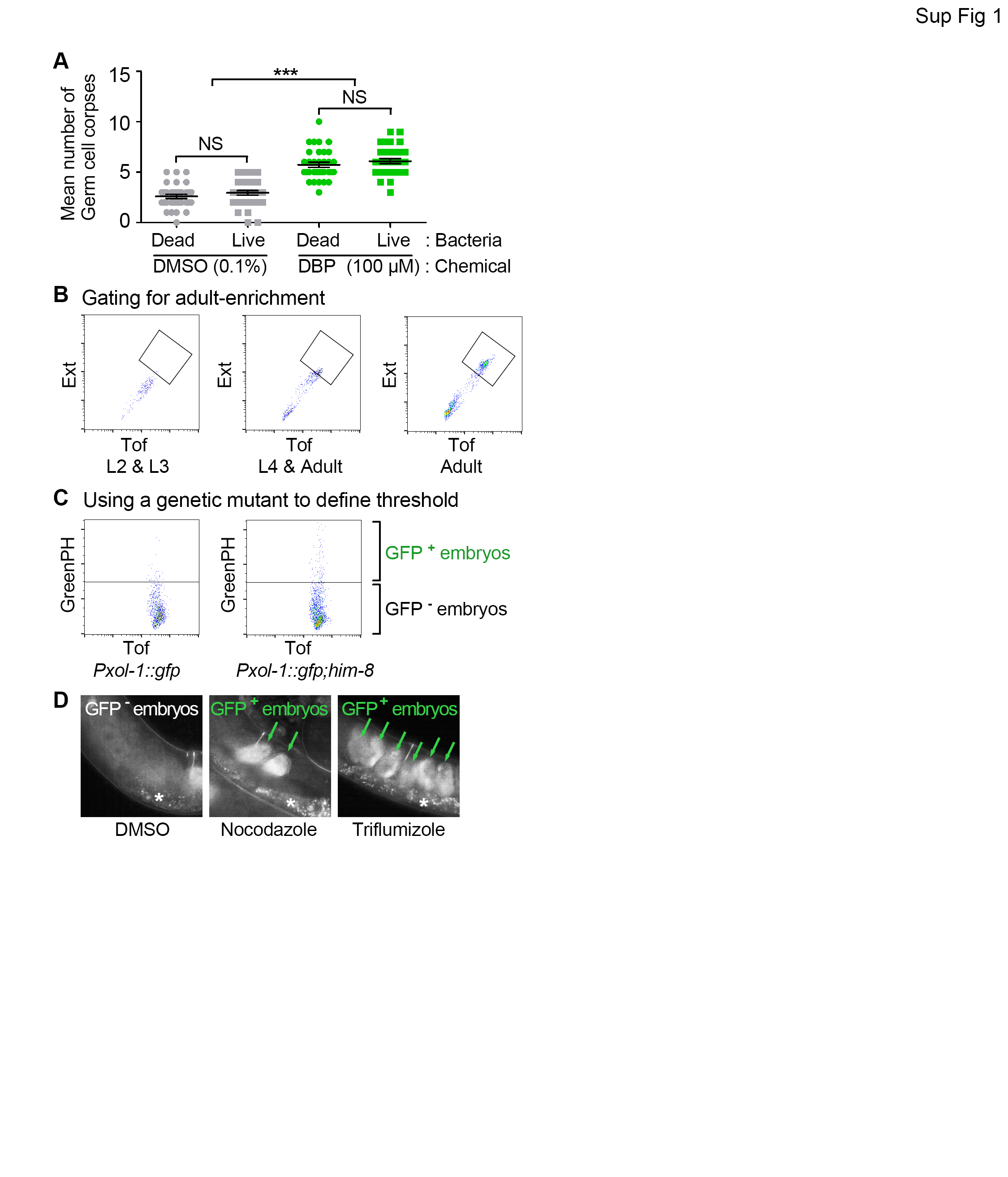

Supplement: S1 Fig — (A) Mean number of germ cell corpses detected for DMSO (0.1%) and DBP (100 μM) exposed worms grown in the presence of either live or heat inactivated (dead) OP50 E. coli. There were no differences detected within DMSO or DBP groups (NS), demonstrating that the effects were not due to the live bacteria (i.e. their response to the chemical stressor) in the media. Analysis was done for two independent biological repeats. More than 30 gonads were scored for each chemical. Error bars represent SEM. ***P<0.0001 by the two-tailed Mann-Whitney test, 95% C.I. (B) L2, L3, L4 and adult populations were sorted through the COPAS Biosort and used to draw a gate capturing only the adult population. The population in this gate was measured for GFP fluorescence intensity. ToF, time-of-flight. (C) Two genetic mutants, Pxol-1::gfp and Pxol-1::gfp;him-8, were used to define the threshold for GFP+ embryos, GFP- embryos and debris. Reading parameters used were ToF for the x-axis and GFP peak height for the y-axis. (D) The induction of X chromosome nondisjunction can be visualized by fluorescence microscopy. GFP+ embryos are visible within the chemical treated worm’s uterus (arrows). Two chemicals that elicit increased chromosome nondisjunction, nocodazole, a microtubule disruptor, and triflumizole, a pesticide [15], were used as positive controls. Asterisks indicate gut autofluorescence. (TIF) [file pgen.1007975.s001.tif]

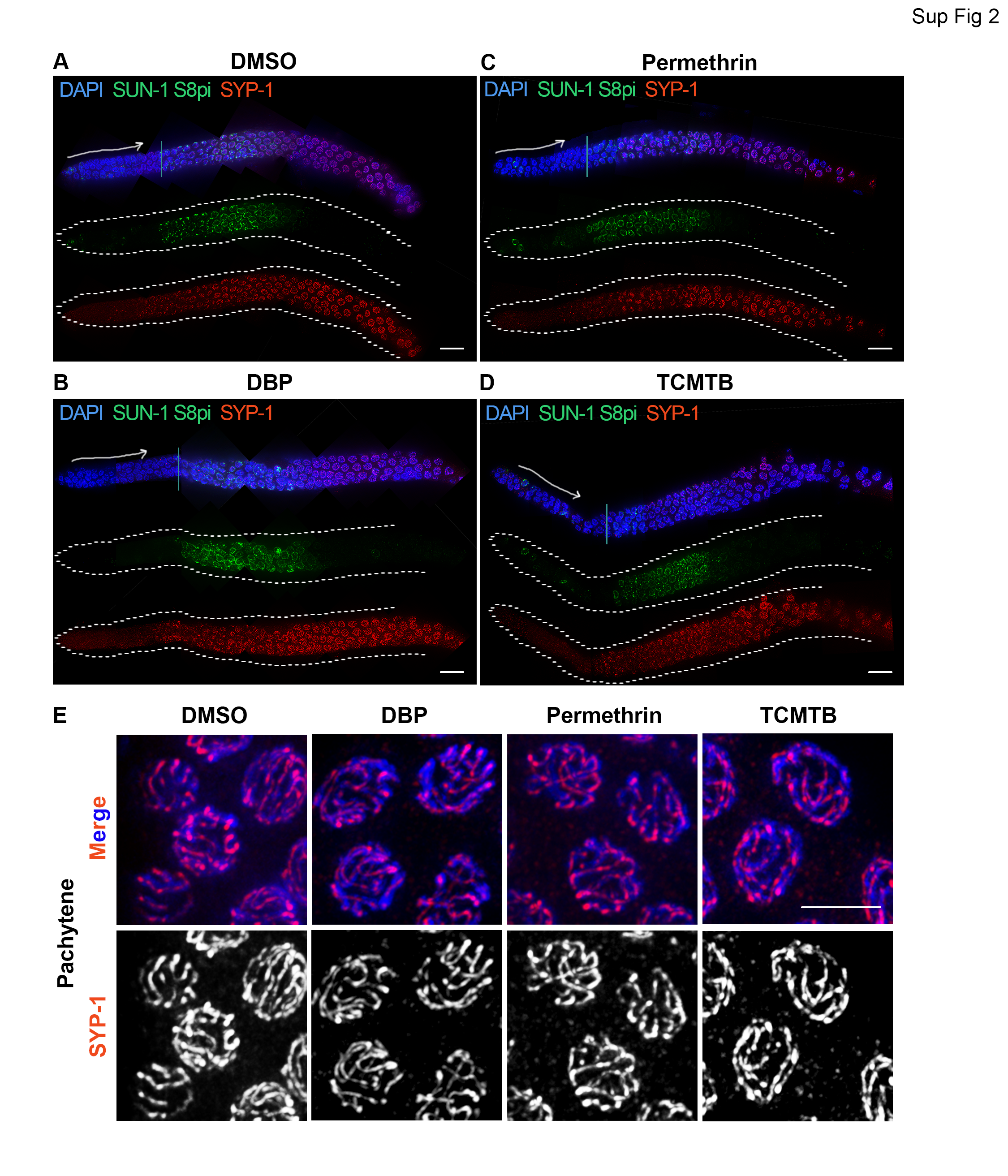

Supplement: S2 Fig — (A-D) Low magnification images of whole mounted gonads immunostained for SYP-1 (red), a central region component of the synaptonemal complex (SC), and phosphorylated SUN-1 (SUN-1 S8pi; green), a marker for progression of early prophase I events. All three chemical exposures exhibit normal timing of SC assembly and disassembly and normal duration of SUN-1 S8 signal compared to vehicle. Dissected gonads are oriented from left to right as indicated by the white arrows. Blue vertical bar indicates entrance into meiosis. Gonads are outlined to facilitate visualization. Scale bars, 10 μm. (E) High magnification images of mid pachytene nuclei from whole-mounted gonads immunostained for SYP-1 (red) and co-stained with DAPI (blue). Continuous full tracks of SYP-1 were observed between homologs in mid pachytene. Scale bar, 5 μm. (TIF) [file pgen.1007975.s002.tif]

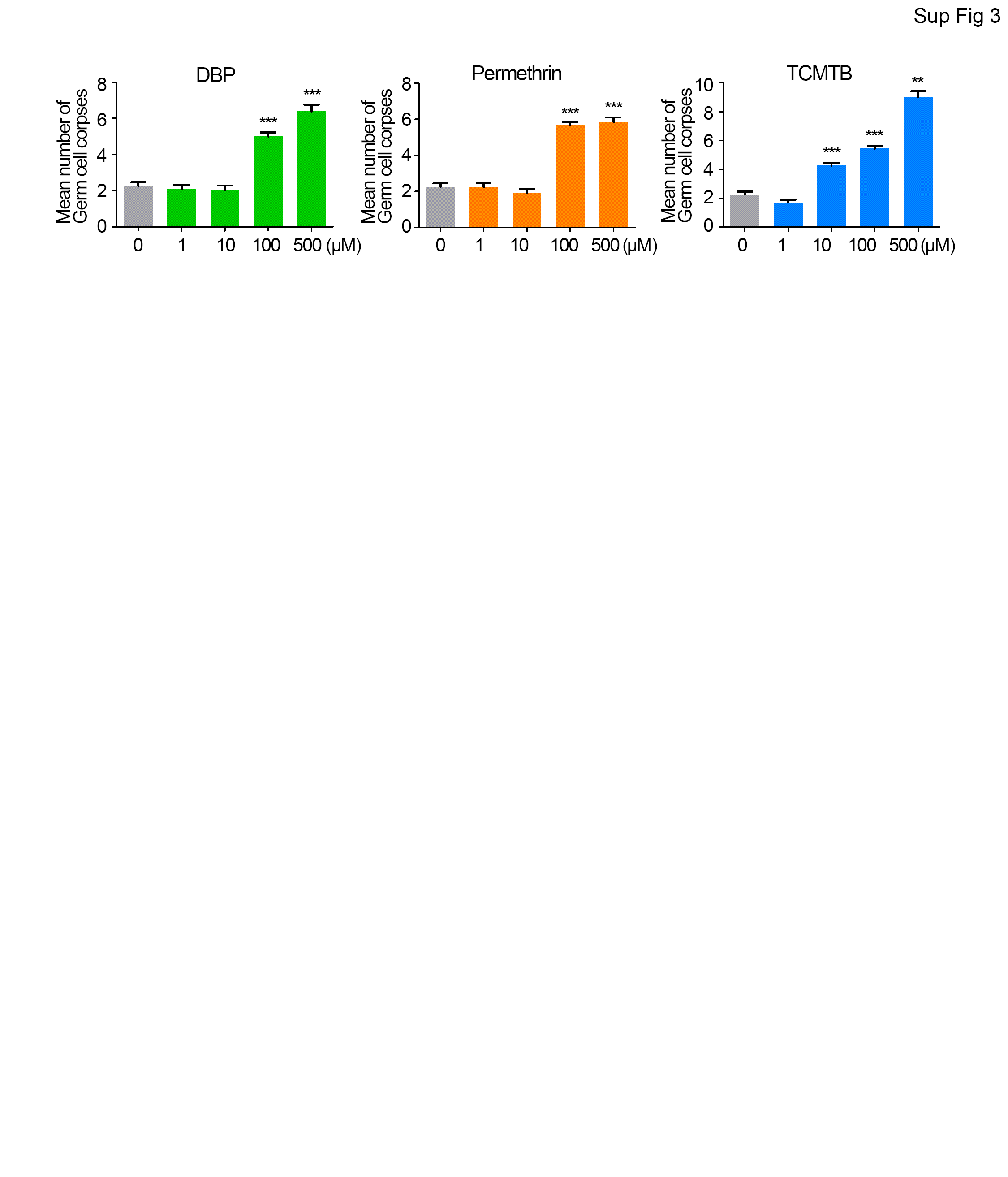

Supplement: S3 Fig — Chemical exposures caused a significant increase in the number of germ cell corpses observed at late pachytene compared to vehicle alone starting at 100 μM for DBP and permethrin and at 10 μM for TCMTB. Germ cell corpses from more than 30 gonads were scored for each chemical exposure. Error bars represent SEM. **P<0.01, ***P<0.0001 by the two-tailed Mann-Whitney test, 95% C.I. (TIF) [file pgen.1007975.s003.tif]

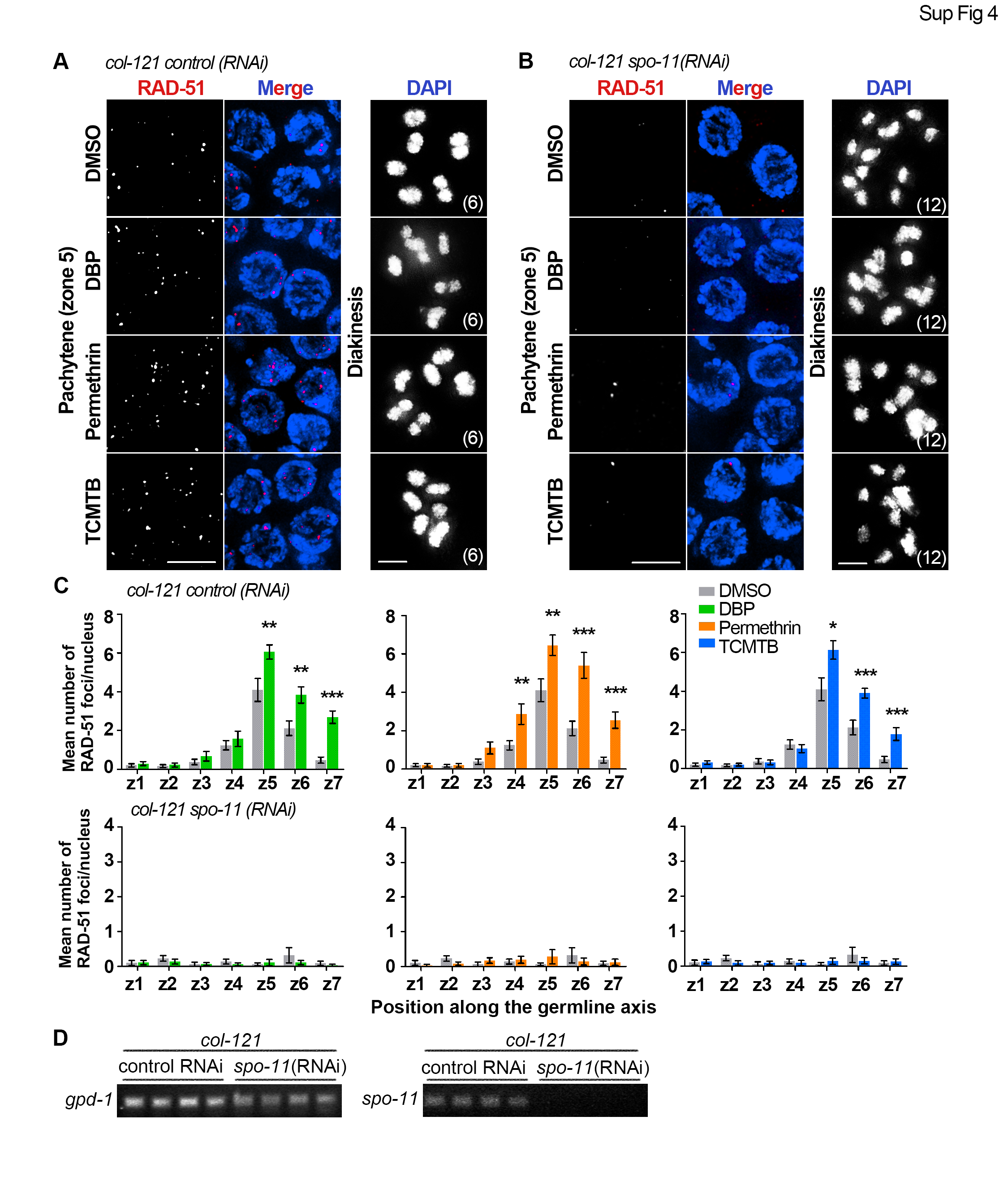

Supplement: S4 Fig — (A, B) Representative images of pachytene nuclei (z5) immunostained for RAD-51 (red), co-stained with DAPI (blue) and high magnification images of oocytes at diakinesis positioned right before the spermatheca (-1 oocyte) in col-121 control (empty vector) RNAi (A) or col-121 spo-11(RNAi) (B). Six bivalents are observed in 100% (n = DMSO: 11, DBP: 10, permethrin: 25, and TCMTB: 18) of the oocytes scored in control RNAi. In contrast, 12 univalents are observed in 100% (n = DMSO: 28, DBP: 29, permethrin: 25, and TCMTB: 22) of the -1 oocytes upon SPO-11 depletion indicating that RNAi depletion worked effectively. While elevated levels of RAD-51 foci are observed in pachytene nuclei for all indicated chemical exposures compared with control, scarcely any RAD-51 foci are observed in pachytene nuclei for either the chemical exposures or vehicle alone when SPO-11 is depleted. Scale bar, 5 μm. (C) Histograms show the mean number of RAD-51 foci scored per nucleus for each zone from col-121 control(RNAi) (C) and col-121 spo-11(RNAi) worms (D). Three gonads from two independent biological repeats were scored for each indicated exposure. Error bars represent SEM. *P<0.05, **P<0.01, ***P<0.001 by the two-tailed Mann-Whitney test, 95% C.I. (D) RT-PCR of col-121 spo-11(RNAi) compared to col-121 control(RNAi) (empty vector). Each lane corresponds to a single worm lysate and indicates the effective depletion of spo-11 by RNAi (shown are the single worm lysates from vehicle alone). gpd-1 expression was used as a loading control. (TIF) [file pgen.1007975.s004.tif]

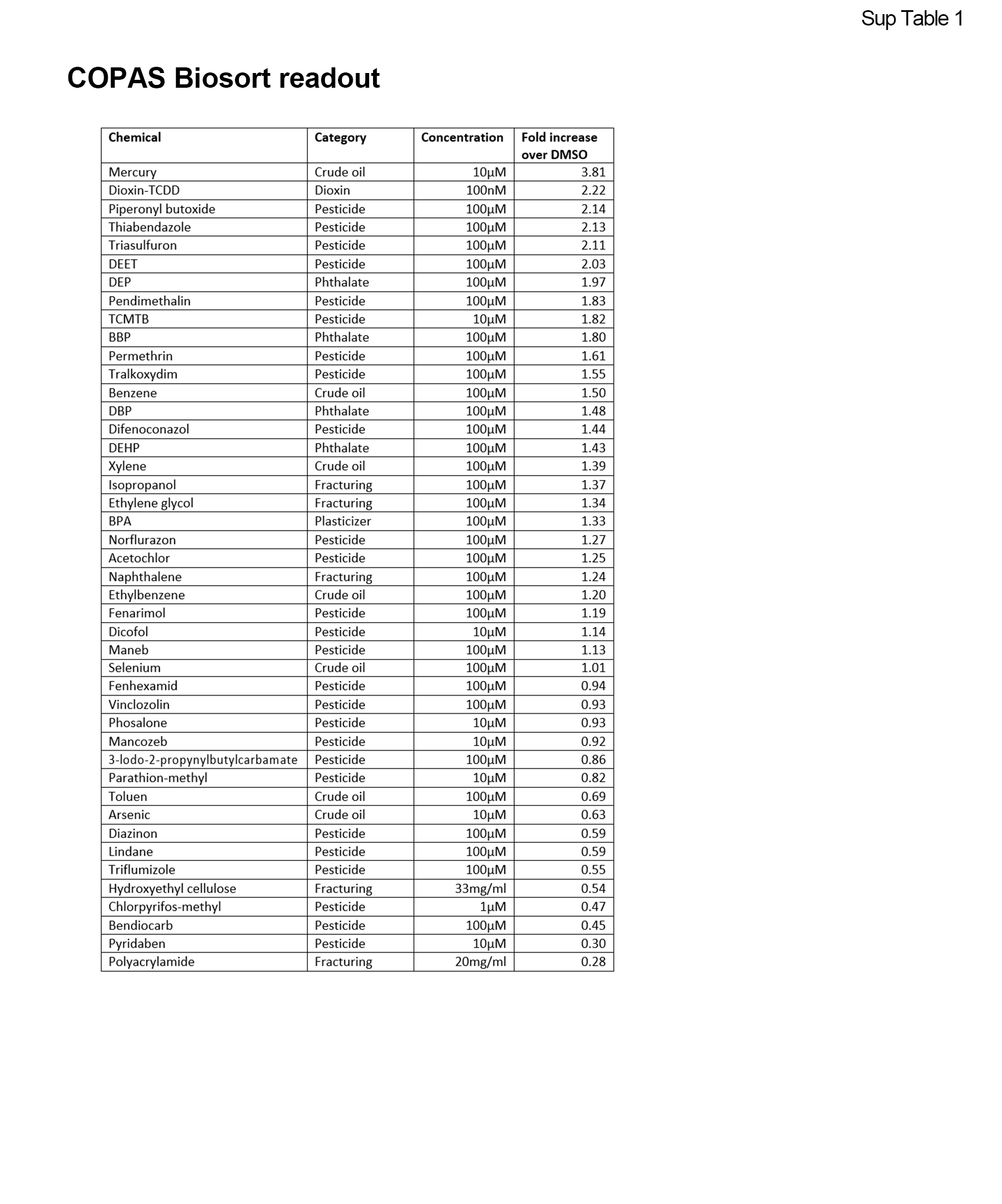

Supplement: S1 Table — Categories indicate the class or use of the chemicals tested (pesticide, phthalate, crude oil processing and hydraulic fracturing; exceptions are TCDD and BPA listed as dioxin and plasticizer, respectively). Concentrations assessed for each chemical in the high-throughput screen are indicated (all chemicals were diluted in DMSO). Chemicals are ranked based on the fold increase in GFP+ embryos detected compared with DMSO-treated (vehicle alone) worms. A minimum of 5,000 worms were screened for each chemical. (TIF) [file pgen.1007975.s005.tif]
